# Peroxisome Proliferator-Activated Receptor-*γ* Ligands: Potential Pharmacological Agents for Targeting the Angiogenesis Signaling Cascade in Cancer

**DOI:** 10.1155/2008/431763

**Published:** 2008-04-23

**Authors:** Costas Giaginis, Anna Tsantili-Kakoulidou, Stamatios Theocharis

**Affiliations:** ^1^Department of Forensic Medicine and Toxicology, Medical School, University of Athens, 11527 Athens, Greece; ^2^Department of Pharmaceutical Chemistry, School of Pharmacy, University of Athens, Panepistimiopolis, Zografou, 15771 Athens, Greece

## Abstract

Peroxisome proliferator-activated receptor-*γ* (PPAR-*γ*) has currently been considered as molecular target for the treatment of human metabolic disorders. Experimental data from in vitro cultures, animal models, and clinical trials have shown that PPAR-*γ* ligand activation regulates differentiation and induces cell growth arrest and apoptosis in a variety of cancer types. Tumor angiogenesis constitutes a multifaceted process implicated in complex downstream signaling pathways that triggers tumor growth, invasion, and metastasis. In this aspect, accumulating in vitro and in vivo studies have provided extensive evidence that PPAR-*γ* ligands can function as modulators of the angiogenic signaling cascade. In the current review, the crucial role of PPAR-*γ* ligands and the underlying mechanisms participating in tumor angiogenesis are summarized. Targeting PPAR-*γ* may prove to be a potential therapeutic strategy in combined treatments with conventional chemotherapy; however, special attention should be taken as there is also substantial evidence to support that PPAR-*γ* ligands can enhance angiogenic phenotype in tumoral cells.

## 1. INTRODUCTION

Angiogenesis, the development of new capillaries from preexisting microvessels, plays a crucial role in several normal physiological processes, such as embryonic development, ovulation, wound healing, as well as tissue and organ regeneration. Angiogenesis also constitutes a crucial step in the aetiology of diverse pathological states, including cancer, diabetic retinopathy, age-related macular degeneration, psoriasis, and rheumatoid arthritis [1, 2]. In the last few years, the complicated biochemical mechanisms governing neovessel formation have been well established. These include the proliferation of endothelial cells (ECs) from preexisting capillaries, the breakdown and reassembly of the extracellular matrix (ECM) and the morphogenic process of endothelial tube formation [2, 3]. Numerous growth factors, including vascular endothelial growth factor (VEGF) family, basic fibroblast growth factors (bFGFs), platelet-derived growth factor (PDGF), hepatocyte growth factor (HGF), placenta growth factor (PGF), matrix metalloproteinases (MMPs), ephrin family, angiopoetin-1 (Ang-1), interleukins (IL-2, -6, -8), as well as various endothelial surface molecules such CD31, CD34, CD36, CD144, and a_v_b_3_ integrins, have been found to control essential steps within angiogenesis process [1–3]. The generation and release of antiangiogenic factors, such as interferon (INF) -*α*, -*β*, -*γ*, platelet factor 4 (PF4), and tissue inhibitors of MMPs (TIMPs) contribute to the coordinated downregulation of the angiogenic process within physiologic angiogenesis [4].

Peroxisome proliferator-activated receptors (PPARs) are members of the nuclear hormone receptor superfamily of ligand-activated transcription factors and include three different isotypes: PPAR-*α*, PPAR-*β*/*δ*, and PPAR-*γ* [5, 6]. PPAR-*γ*, the most extensively studied amongst them, functions as ligand-activated transcription factor by binding to specific DNA sequences, termed to as peroxisome proliferator response elements (PPREs), in the promoter of the target genes only as a heterodimer with the retinoid X receptor (RXR) [7–9]. PPRE has been mainly identified in the upstream regulatory sequences of genes related to metabolic pathways [7–9]. In addition, recent studies have revealed that PPAR-*γ* can regulate gene expression independently of PPRE, either by suppressing growth hormone protein-1 (GHP-1), a transcription factor involved in pituitary specific gene expression, or by interfering with the function of activator protein-1 (AP-1), signal transducer and activator of transcription-1 (STAT-1) and nuclear factor-*κ*B (NF-*κ*B) [7, 10–12]. In this context, the identification of a sumoylation-dependent pathway by which PPAR-*γ* represses transcriptional activation of inflammatory response genes has recently been reported [13]. This mechanism provides a possible explanation for how ligand-bound PPAR-*γ* activation can be converted from an activator of transcription to a promoter-specific repressor of NF-*κ*B target genes [13].

A wide range of natural and synthetic structurally diverse compounds has been reported as potent PPAR-*γ* ligands. The long chain polyunsaturated fatty acids and their derivatives, such as 15-deoxy-Δ^12,14^-prostaglandin J_2_ (15d-PGJ2), as well as nitrolinoleic acids are known natural occurring PPAR-*γ* ligands [14, 15]. Recently, curcumin, a well-documented anticancer phytochemical component of turmeric, has been shown to exert anti-inflammatory functions via upregulation of PPAR-*γ* activation [16]. Thiazolidinediones (TZDs) and tyrosine-based derivatives, such as glitazars (tesaglitazar, farglitazar), constitute the most well-known synthetic ligands [17, 18], while relatively lower binding affinity for PPAR-*γ* has also been reported for some nonsteroidal anti-inflammatory drugs (NSAIDs) [19]. TZDs represent a promising class of oral antidiabetic agents, some of which are already marketed drugs (pioglitazone-PGZ and rosiglitazone-RGZ) for the treatment of type II diabetes mellitus [20]. Interestingly, a wide spectrum of action for TZDs beyond the treatment of diabetes, including anti-inflammatory and antineoplastic properties, as well as targeting signaling pathways implicated in atherosclerosis and osteoporosis has been reported [21–23]. In the last decade, more than 1000 PPAR-*γ* ligands belonged to several distinct chemical classes have been synthesized and evaluated for their binding and transactivation to their receptor. In this aspect, screening drug-like characteristics in the chemical space of PPAR-*γ* ligands have currently been considered as an emerging demand in the aim to discover more potent compounds with improved absorption, distribution, metabolism, excretion/toxicity (ADME/Tox) properties, avoiding potential toxic side effects, as well as pharmacokinetic and pharmacodynamic problems [24, 25].

To date, there has been a substantial accumulation of evidence that PPAR-*γ* ligands exert regulatory effects on angiogenesis process related to diverse disease states, including cancer and diabetes [26–28]. It is also well documented that they directly affect tumor cells by inhibiting cell growth and inducing cell differentiation and apoptosis in various cancer types [21, 29, 30]. In view of the fact that angiogenesis is implicated in tumor development and metastasis and its inhibition could serve as potent antitumor side-therapeutic approach, the current review summarizes the latest knowledge of the role of PPAR-*γ* ligands in angiogenesis related to cancer, highlighting in the underlying mechanisms.

## 2. ANGIOGENESIS IN CANCER

Tumor angiogenesis constitutes an essential component of tumor growth, invasion, and metastasis that depends on a net balance of angiogenic and antiangiogenic mediators, which are secreted by both tumor and host infiltrating cells [31]. Currently, it is well established that this dynamic balance between angiogenic stimulators and inhibitors, controls the angiogenic signaling cascade governing the transformation of a tumor from a nonangiogenic to an angiogenic phenotype [32]. The acquisition of angiogenic phenotype has been considered as a rate-limiting step in tumor progression, which allows the tumor to transform from a small lesion to a rapidly expanding mass with metastatic potency [33]. On the other hand, human tumors arise in the absence of angiogenic activity and may exist in a microscopic dormant state for months to years without neovascularization [34]. In this context, hypoxia, developed within rapidly proliferating tissues or as a result of the occlusion of blood vessels, has been considered as a primary physiological regulator of the angiogenic switch [35]. The key mediators of this response are members of the hypoxia-inducible factor (HIF) family of proteins that function as transcriptional regulators, stimulating the expression of a multitude of genes important for oxygen homeostasis [36, 37]. In addition, HIF has been found to enhance the expression of several angiogenic mediators, including VEGF-R1, VEGF-R2, Ang-1, Ang-2, MMP-2, and MMP-9 in malignant tumors [36, 38].

In response to hypoxia, tumor cells turn on the angiogenic signaling cascade by secreting various potent angiogenic mediators, such as VEGF, PDGF, bFGFs, angiopoetins, HGF, fibronectin, and heparanase that in turn activate endothelial cells of preexisting capillaries to produce MMPs for the collapse of ECM [39]. Degradation of ECM by MMPs allows endothelial cells to migrate in response to chemotactic growth factors, including VEGF, PDGF, and bFGFs [33, 39]. Members of CXC chemokine family, such as IL-2, -6, -8, and integrins *α*_v_ *β*_3_, are also involved in the angiogenic cascade. It should be noted that in the case of high progressive tumors, the release of endogenous antiangiogenic factors are insufficient to counteract the net effect of angiogenic ones. Thus, the formation of new blood vessel is formed after attracting accessory cells, mainly pericytes and smooth muscle cells, producing a new basement membrane and a firm ECM [39, 40]. The above-mentioned angiogenic mediators have been joined by others including Notch/Delta, semaphorin, ephrin, and roundabout/slit families of proteins [40]. Besides this, blockage of NF-*κ*B activity has been shown to reduce VEGF gene expression in highly malignant tumor cells, since a binding site for this transcription factor has been identified within the VEGF promoter [41]. Each of the sequential steps within angiogenic cascade could be considered as a potential single target for the development of new drug candidates against tumor vasculogenesis.

Currently, numerous therapeutic approaches have been designed in the aim to control tumor angiogenic cascade by targeting the above-mentioned angiogenic mediators [40, 42]. In this context, more than a few angiogenesis inhibitors have already been approved for the treatment of cancer, while several compounds are in the late stage of clinical trials. The main category of the antiangiogenic compounds exerts its action indirectly either by neutralization of tumor-derived angiogenic factors or preventing the receptors/signaling pathways of these growth factors. In this regard, VEGF isoforms and their tyrosine kinase receptors VEGFRs, as well as epidermal growth factor (EGF) and its receptor (EGFR) are currently explored in clinical trials as drug candidates against cancer [43–45].

With respect to angiogenesis inhibitors, several angiostatic compounds, such as endostatin, thrombospodin-1 (TSP-1), tumstatin, angiostatin, and 16-kDa N-terminal fragment of human prolactin (16K hPRL) have been reported to directly and selectively suppress endothelial cell migration inducing EC apoptosis and cell cycle arrest within tumor neovascularization [46, 47]. It should be mentioned that most of these angiostatic compounds are also naturally occurring molecules that compensate with angiogenic factors in order to control angiogenic cascade in normal physiologic conditions. In addition, targeting MMPs by such agents has been reported, underlining the importance of ECM remodeling during angiogenesis process. Activation of NF-*κ*B may also be a possible mechanism of such angiostatic agents to induce EC apoptosis and to improve immune response within angiogenesis process [46, 47].

## 3. INHIBITION OF ANGIOGENESIS BY PPAR-*γ* LIGANDS

**Figure 1:**
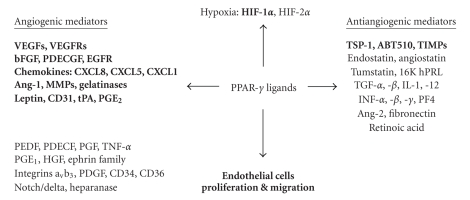
The network of the components implicated in the angiogenesis process in cancer and the impact of PPAR-*γ* ligands illustrated by blue color.

PPAR-*γ* ligands can regulate tumor angiogenesis via direct effects on ECs proliferation and migration and/or through indirect mode of action by affecting the counterbalance between angiogenic and antiangiogenic mediators (Figure 1, Table 1).

### 3.1. Direct effects on endothelium

PPAR-*γ* has been reported to be expressed in endothelial cells and PPAR-*γ* ligands are well established to exert direct effects on them [48, 49]. PPAR-*γ* activation by either naturally occurring or synthetic ligands resulted in potent inhibition of growth factor-induced differentiation and proliferation in human umbilical vein endothelial cells (HUVECs) and choroidal endothelial cells (CECs) [48, 49]. In this regard, PPAR-*γ* dependent mode of action has been shown to stimulate caspase-mediated ECs apoptosis [50]. Importantly, RGZ levels able to inhibit ECs proliferation are readily achieved in patients undergoing standard antidiabetic RGZ treatment [51]. Moreover, both RGZ and PGZ, at relative pharmacological concentrations, resulted in a strong prevention of VEGF-induced tube formation and ECs migration [52, 53]. Mechanistically, it has been supported that angiogenesis inhibition by RGZ in HUVECs involves a proapoptotic mechanism which includes the implication of the PPAR-*γ*-mediated NO production and the maxi-K channel activation [54]. Maxi-K channels, essential mediators of vascular remodeling and angiogenesis, are synergically regulated by various intracellular second messengers including NO [54]. Hence, a possible proapoptotic mechanism for the PPAR-*γ*-mediated NO production has been suggested [55]. Recently, pigment epithelium-derived factor (PEDF), a potent antiangiogenic glycoprotein, has been shown to stimulate HUVECs apoptosis through sequential induction in the expression and transcriptional activity of PPAR-*γ*. PEDF upregulated p53 expression via PPAR-*γ*, supporting evidence that p53 may be a major target in PPAR-*γ* mediated ECs apoptosis [56].

PPAR-*γ* has also been reported to be expressed in tumor ECs, presenting a relative overexpression in tumor-induced endothelial sprouts compared to normal endothelium. In this case, endothelial and tumoral cells have been shown to display inhibition even at low TZDs doses [57]. Importantly, TZDs inhibited tumor cell invasion across blood vessel endothelium. In fact, RGZ at concentrations close to the range of its binding affinity for PPAR-*γ* [8] exerted inhibitory effects on tumor angiogenesis in malignant cell lines and in immunodeficient mice with transplanted tumors [57]. In this regard, it should be mentioned that RGZ concentrations of 5 *μ*M and higher led to the phosphorylation of eIF-2*α* in HUVECs, supporting evidence that the inhibition of ECs proliferation could also be mediated through a PPAR-*γ* independent pathway. However, at even lower concentration range (0.1–1 *μ*M), at which PPAR-*γ* is activated, RGZ was capable of exerting even stronger antiproliferative effects on ECs in vitro [57]. In this context, the concentration range of PPAR-*γ* ligands should be taken into careful consideration, because over a concentration limit, which may be varied amongst the different types of cells, in vitro, as well as amongst different species, in vivo, receptor-independent actions could be elicited. Such PPAR-*γ* mode of action has recently been reviewed by Feinstein et al., who suggested an alternative mitochondrial target for TZDs, termed as mitoNEET [12]. To this point, it should be noted that higher doses of RGZ were less effective in inhibiting angiogenesis and hence lung metastasis than lower doses that are actually comparable to the serum levels of RGZ in diabetic patients [27, 51]. Overall, although PPAR-*γ* ligands can also induce EC apoptosis as mentioned in the previous paragraph, it is unlikely that they do this under physiological conditions as this may result in a severe thrombosis. Thus, it should be emphasized the fact PPAR-*γ* ligands may target better EC proliferation as shown by Panigrahy et al. [27] and Freed et al. [51].

Orthotopic implantation of H2122 nonsmall cell lung adenocarcinoma cells overexpressing PPAR-*γ* into the lungs of nude mice attenuated tumor growth and metastasis by selective inhibition of invasive metastasis, and activation of pathways that promote a more differentiated epithelial phenotype [73]. This evidence deserves special attention since both angiogenesis and invasion are crucial for the formation of metastasis and the recurrence of tumors. Moreover, reintroduction of exogenous TSP1 or its peptide derivative ABT510 can reverse the angiogenic switch, and thus blocking tumor expansion. TSP-1 is a well-known potent angiogenesis inhibitor that targets ECs for apoptosis through signaling cascade at its receptor CD36. In tumor xenografts, TGZ, RGZ, and 15d-PGJ2 coupled to ABT510 suppressed angiogenesis and induced ECs apoptosis in a CD36 dependent manner [62]. In this context, 15d-PGJ2 treatment upregulated CD36 surface expression in human monocytic cell line THP-1 by enhancement of CD36 gene transcription [74]. Thus, PPAR-*γ* could be considered as a critical regulator of CD36 expression, as both natural and synthetic PPAR-*γ* ligands are capable of increasing CD36 expression [75].

Receptor-mediated effects for PPAR-*γ* ligands in inhibiting angiogenesis through direct mode of action on endothelium seem to be dominated [28, 57]. In this regard, PPAR-*γ* knockout mice embryos died on day 10 of life because of interference with the terminal differentiation pattern of trophoblasts, as well as the loss of vascular development in the placenta [76, 77]. It has also been suggested that PPAR-binding protein (PBP), a coactivator of PPAR-*γ*, may function as a negative modulator of ECs proliferation [77]. Such genetic data provides additional evidence that PPAR-*γ* functions as modulator of angiogenesis; however, receptor-independent action should not be excluded. In this aspect, Artwohl et al. showed PPAR-*γ*-independent antiproliferative effects on HUVECs associated with lactate release, possibly due to inhibition of mitochondrial function [78].

### 3.2. Indirect effects on the net balance between angiogenic and antiangiogenic factors

Beyond the direct mode of action on the endothelium, PPAR-*γ* ligands have been reported to downregulate angiogenesis process via indirect mechanisms by modulating the levels of the endogenous angiogenesis mediators (Figure 1, Table 1). In this context, VEGF/VEGFR signaling pathway seems to be a key target for PPAR-*γ* ligands in inhibiting angiogenesis. Xin at al. provided the first evidence that 15d-PGJ2 reduced VEGFRs m-RNA levels in HUVECs [48]. It has also been supported that PPAR-*γ* ligands may have bifunctional properties in KDR gene expression that involve the enhancement of Sp1-DNA binding in absence of ligand by PPAR-*γ* itself and the suppression of Sp1-DNA-binding in presence of PPAR-*γ* ligands [79]. Moreover, PPAR-*γ* activation has been shown to downregulate leptin and tumor necrosis factor (TNF-*α*), two well-known angiogenesis-inducing factors [80, 81]. In fact, PPAR-*γ* activation by TZDs attenuated leptin gene expression both in vivo and in vitro [82, 83] and blocked leptin-induced ECs migration through inhibition of Akt and eNOS signaling [84]. This evidence suggests that endothelial phosphatase and tensin homologue mutated on chromosome ten (PTEN), a negative regulator of PI3K → Akt signaling, may play a crucial role in the ECs antimigratory actions of TZDs [84].

Tumor-associated angiogenesis has been reported to be indirectly suppressed by blocking the expression of angiogenic stimulators in response to PPAR-*γ* ligand activation. In this regard, PPAR-*γ* activation by TGZ or PGZ diminished the production of the angiogenic ELR + CXC chemokines IL-8 (CXCL8), ENA-78 (CXCL5), and Gro-*α* (CXCL1) in human non-small-cell lung cancer cell line A459 [63]. This effect was ascribed to the negative modulation of NF-*κ*B activation [63]. In addition, CGZ was found to decrease PGE_2_ production through downregulation of cyclooxygenase-2 (COX-2) expression in human non-small-cell lung carcinoma A427 and A549 cell lines [64]. Interestingly, utilization of a dominant negative PPAR-*γ* construct revealed that the effect of CGZ on both COX-2 and PGE2 was mediated through PPAR-*γ* independent pathways [64]. Another study demonstrated that 15d-PGJ2 attenuated the expression of Ang-1 and hence the angiogenic process through the angiopoietin-Tie2 system in the gastric cancer cell line MKN45 [67]. Ang-1 is involved in the regulation of maturation and stabilization of the vascular wall, and thus it might be a potential target for inhibiting tumor angiogenesis. Moreover, in a model of human anaplastic thyroid carcinoma, RS5444, a novel high-affinity PPAR-*γ* agonist exerted potent antiangiogenic action, in vivo, by decreasing CD31, a specific molecular marker of blood vessels [71]. In this regard, PPAR-*γ* ligand treatment (TZDs, 15d-PGJ2, and RS1303) dose-dependently suppressed cell proliferation by inducing apoptosis instead of differentiation in five human anaplastic carcinoma cell lines (MSA, IAA, ROA, K119, and KOA-2) [61]. Recently, CGZ has also been shown to produce antitumor effects against ovarian cancer, in vitro and in vivo, in conjunction with reduced angiogenesis and induction of apoptosis [65]. In this case, CGZ induced antitumor effects were comparable to that of cisplatin and were ascribed to inhibition of VEGF production in relation to PGE_2_ reduction, an endogenous stimulator of angiogenesis and invasiveness [65]. PPAR-*γ* ligands have also been shown to repress VEGF gene expression via a PPAR-*γ*-responsive element (PPRE) in the VEGF gene promoter in both primary and transformed human endometrial cell cultures [60]. This study provided substantial evidence that PPAR-*γ* ligands may be exploited pharmacologically to inhibit pathological vascularization in complications of pregnancy, endometriosis, and endometrial adenocarcinoma [60].

As mentioned in Section 3.1, RGZ suppressed tumor angiogenesis by direct mode of action in endothelium; however, indirect antiangiogenic effects have also been reported [57]. More to the point, RGZ, at low doses, in vitro, inhibited bovine capillary ECs and reduced VEGF production by tumor cells [57]. RGZ also suppressed angiogenesis in the chick chorioallantoic membrane, in the avascular cornea, in vivo, as well as in a variety of primary tumors, such as glioblastoma U87 and Lewis lung carcinoma cells, in vitro [57]. Likewise, both PGZ and 15d-PGJ2 have been shown to inhibit, dose- and time-dependently, VEGF and bFGF secretion in human renal cell carcinoma cells [66]. Importantly, antiangiogenic effects were observed at the dose of 5 *μ*M PGZ, a level that is also obtained in diabetic patients after standard PGZ treatment [66]. On the other hand, there is nonavailable data so far concerning the effect of PPAR-*γ* ligand treatment on the expression and/or secretion of antiangiogenic mediators. In this regard, future studies focused on the impact of PPAR-*γ* ligands in mediators, such as endostatin, TSP-1, tumstatin, angiostatin, and 16K hPRL are strongly recommended.

Angiogenesis constitutes a crucial step for tumor invasion and formation of metastasis. In this aspect, PPAR-*γ* ligand treatment attenuated the invasiveness of pancreatic tumor cells, reducing MMP-2 and -9 protein levels and activity [70]. Moreover, the secretion of the invasive factor tissue plasminogen activator (tPA) was decreased by RGZ treatment in pancreatic tumor AsPC-1 cells through receptor mediated mechanisms [58]. Treatment of the highly aggressive human breast cancer cell line MDA-MB-231 with synthetic and natural PPAR-*γ* ligands, at noncytotoxic concentrations, also resulted in a significant inhibition of the invasive capacity [59]. In fact, TIMP-1 was upregulated by PPAR-*γ* ligand treatment, while the gelatinolytic activities of gelatinases in the conditioned media were decreased [59]. Moreover, PPAR-*γ* ligands downregulated the invasive potential of anaplastic thyroid carcinoma cells, and this effect was prominent in 3 cell lines, which exhibited higher expression level of the PPAR-*γ* gene or protein [61].

Clinical evidence from a pilot study enrolled 6 patients with angiosarcoma and hemangioendothelioma, revealed that the angiostatic triple combination of PGZ, rofecoxib, and metronomic trofosfamide exhibited high efficacy in the palliative care of patients [85]. Until this study, antiangiogenic drugs such PGZ and rofecoxib had not been considered for the treatment of human angiosarcoma. In support of this view, a case report study has demonstrated that this novel antiangiogenic therapy was effective in a patient with endemic Kaposi sarcoma and led to partial remission that was stable for 18 months without significant side effects [86]. Hence, targeting PPAR-*γ* may prove to be a potential therapeutic strategy in combined treatments with conventional chemotherapy for patients with vascular disorders [87].

## 4. INDUCTION OF ANGIOGENESIS BY PPAR-*γ* LIGANDS

The most comprehensive data so far render PPAR-*γ* ligands as potent inhibitors of angiogenesis; however, there are several lines of evidence to support that PPAR-*γ* ligand activation can also trigger angiogenic cascade (Table 1). In fact, increased VEGF mRNA levels and induction of angiogenesis in response to PPAR-*γ* ligands treatment have been reported both in vitro and in vivo [88–91]. Interestingly, TZDs have been considered as potential pharmacological agents for angiogenesis induction in the treatment of ischemic artery disease [89]. Recent clinical evidence has also demonstrated that RGZ treatment improved endothelial progenitor cell (EPC) number and migratory activity in diabetic patients [92, 93]. In addition, PGZ treatment was found to improve endothelial function by increasing the number and the migratory capacity of EPCs in animal and human studies [94, 95]. Another study has revealed that eNOS upregulation induced by RGZ may be the dominant mechanism through which RGZ enhanced angiogenesis [91]. However, Gensh et al. did not observe upregulation of vascular eNOS mRNA expression or setback of the PGZ-induced increase of EPCs in the presence of 1-NAME, a NOS inhibitor [94]. These authors suggested that TZDs may regulate EPCs by a mechanism independent of eNOS [94]; however, further studies based on pharmacologic blocking or knockout modeling of eNOS are strongly recommended in order for precise conclusion to be drawn. Importantly, taking into account the discrepancy in literature, Gensh et al. assumed that TZDs may play a double-edged role in angiogenesis signaling by promoting the number and migration of EPCs at lower tissue concentrations obtained by systematic treatment, whereas the antiangiogenic effects are elicited at higher local concentrations [94]. This major remark has also been reported in the case of breast cancer cells where low concentration of PPAR-*γ* ligands increase cell proliferation in contrast to the higher concentrations that suppress cell growth [96]. The urgent demand to define and monitor the dosage of PPAR-*γ* ligands in clinical trials for cancer therapy is thoroughly discussed by Panigrahy et al. [27]. In this aspect, special attention deserves the fact that atorvastatin, a 3-hydroxy-3-methylglutaryl coenzyme A (HMG-CoA) reductase inhibitor, and PGZ increased myocardial 15d-PGJ2 levels in the rat myocardium and HUVECs [97]. 15d-PGJ2 was produced mainly via COX-2 and activated PPAR-*γ*. Interestingly, it was supported that PPAR-*γ* activation was exclusively mediated by 15d-PGJ2 in the case of atorvastatin, whereas PGZ activated directly PPAR-*γ* or indirectly via 15d-PGJ2 [97]. Thus, these recent findings raise the question whether the final effect of PPAR-*γ* ligands is completely ascribed to the dose of PPAR-*γ* ligand treatment or in addition to the induction of endogenous PPAR-*γ* activators, such as 15d-PGJ2. It should also be taken into account the fact that endogenous nitrated fatty acids that comprise a class of nitric oxide-derived, PPAR-*γ* dependent and cell signaling mediators can modulate systematic inflammatory responses within physiological concentration ranges [98].

There is also substantial evidence, which suggests that PPAR-*γ* ligands stimulate tumor angiogenesis. In this context, 15d-PGJ2 treatment was found to dose-dependently increase the VEGF mRNA expression in both human androgen-independent PC-3 prostate and 5637 urinary bladder carcinoma cells [68]. In addition, 15d-PGJ2 resulted in upregulation of VEGF expression through the induction of heme oxygenase (OH)-1 ERK1/2 phosphorylation in human breast cancer MCF-7 cells, thus contributing to increased angiogenesis in this type of tumor cells [69]. Nimesulide, a selective COX-2 inhibitor, although at relatively high concentrations, enhanced VEGF secretion from pancreatic cancer cells in vitro, as well as from both COX-2-positive and COX-2-negative pancreatic tumors through PPAR-*γ* activation [72]. Importantly, in the case of COX-2-negative pancreatic tumors, nimesulide-stimulated VEGF production was considerably associated with enhanced angiogenesis and tumor growth [72]. Besides this, VEGF was differentially increased, according to the differentiation state of the cells, by the three PPAR isotypes, -*α*, -*β*/*δ*, and -*γ*, in two different human urinary bladder cancer cell lines, RT4 and T24, derived from grade-I and grade-III tumors, respectively [99]. The PPAR ligand-induced VEGF expression seemed to be PPAR-specific and involved an indirect mechanism requiring an intermediary regulatory protein through the MAP (ERK1/2) kinase pathway, probably by a modulation of the phosphorylation state of PPARs [99]. Immunohistochemical analysis in human bladder tumor specimens also revealed statistically significant associations between PPAR-*γ* and several angiogenic factors, such as VEGF, bFGF, platelet-derived endothelial cell growth factor (PDECGF), and EGFR in respect to the incidence of tumor recurrence or progression [100]. On the other hand, no statistically significant differences were observed between PPAR-*γ* immunoreactivity and angiogenesis parameters in skin cancer, whereas the microvessel density was significantly higher in actin keratosis and squamous cell carcinoma that expressed PPAR-*β*/*δ* [101]. These clinical data on PPAR-*γ*-induced signaling implicated in the expression of crucial angiogenic factors in human neoplasia may unfold the development of new therapeutic approaches in those types of cancer in which excessive angiogenesis represents a negative prognostic factor.

## 5. THE IMPACT OF PPAR-*γ* LIGANDS IN HYPOXIA- ASSOCIATED SIGNALING PATHWAYS

As hypoxia is a key regulator of the angiogenic switch, hypoxia-induced angiogenesis is gaining gradually increasing interest as a potential target for cancer therapy. In human bladder tumors and cell lines, several components of the hypoxia response pathway, including HIF-1*α* and HIF-2*α* have been considered as important cofactors of the regulation of VEGF [102]. Recent findings have revealed that PPAR-*γ* can modulate arterial remodeling associated with hypoxic hypertension [103]. In fact, RGZ was found to attenuate and reverse pulmonary arterial remodeling and neomuscularization in rats subjected to chronic hypoxia [104]. Decreased pulmonary arterial (PA) remodeling in RGZ-treated animals was associated with decreased smooth muscle cell proliferation, decreased collagen and elastin deposition, and increased matrix MMP-2 activity in the PA wall [104]. In this aspect, PPAR-*γ* mRNA levels were found significantly lower in human adhesion fibroblasts compared to normal ones in response to hypoxia [105]. Moreover, hypoxia has demonstrated to reduce the mRNA levels of PPAR-*γ* protein in human proximal renal tubular epithelial cells (HPTECs). However, knockout of HIF-1*α* with its dominant negative form did not block the hypoxia-induced reduction in PPAR-*γ* expression [106]. In this regard, substantial evidence has revealed that 15d-PGJ2 can modulate the activities of several transcriptional factors, such as NF-*κ*B and AP-1, including also HIF-1 [107]. The regulation of the aforementioned redox-sensitive transcription factors by 15d-PGJ2 was not necessarily mediated via PPAR-*γ* activation, but rather involves covalent modification or oxidation of their critical cysteine residues acting as a redox sensor [107]. Overall, targeting hypoxia-induced angiogenesis by PPAR-*γ* ligands may prove to be a promising therapy for the treatment of cancer; however, the precise mechanisms involved in hypoxia-induced angiogenesis process remain to be clarified.

## 6. CONCLUSION

At the present, there is quite a lot of evidence to support that PPAR-*γ* may be considered as therapeutic target for diverse disease states in which excessive angiogenesis is implicated, including cancer. The most comprehensive data so far have revealed that PPAR-*γ* ligands are capable of inhibiting angiogenesis implicated in tumor malignant transformation and expansion. Targeting ECs proliferation and migration seems to be a dominant effect of PPAR-*γ* ligands on tumor angiogenesis. Indirect mechanisms that involve the counterbalance between a multitude of endogenous angiogenic and antiangiogenic factors further account for the inhibitory effects of PPAR-*γ* ligands on tumor angiogenesis. According to these data, PPAR-*γ* ligands may unfold new perspectives in clinical use against primary tumor growth and metastasis, since tumors that exhibit multidrug resistance are effectively targeted by antiangiogenic chemotherapy. Such perspectives could be clinically relevant, as PGZ and RGZ are orally administered FDA-approved drugs, already been used by million patients undergoing standard antidiabetic treatment.

On the other hand, there are several lines of evidence that PPAR-*γ* ligands can also enhance tumor angiogenesis progression under certain conditions. This controversy could be attributed to the pleiotropic action of PPAR-*γ* ligands, possibly via cofactors, either coactivators or corepressors. Such discrepancies may also be ascribed either to differences in time and dose of PPAR-*γ* ligand treatment, or to differences among the various organisms and types of cells that have been studied. It should be taken into account that angiogenesis is a multifaceted process that involves a wide range of mediators capable of inducing or suppressing angiogenesis in addition to the degree of tissue hypoxia. Consequently, the final outcome is difficult to be assessed accurately and depends significantly on experimental models and/or treatment conditions. Moreover, each type of cancer in humans presents individual and distinct vascular pattern on the microenvironment in which it is located. Thus, it should be taken into careful consideration the type of cancer being treated when deciding an appropriate therapeutic strategy.

In this aspect, the use of different cancer models, in vitro and in vivo, are strongly recommended to further define the molecular interactions amongst PPAR-*γ*, angiogenic/antiangiogenic factors, and tumor progression markers within the distinct cancer types. Future research effort should also be orientated to the clinical evaluation of PPAR-*γ* expression in aggressive tumor cancers in which various angiogenic/antiangiogenic factors exhibit high prognostic value. Such studies could delineate the potential of PPAR-*γ* ligands in future anticancer therapeutic strategies, either alone or combined with conventional chemotherapy.

## Figures and Tables

**Table 1:** Effects of PPAR-*γ* ligands on tumor angiogenesis.

PPAR-*γ* ligands	Type of cells/organisms	Effects	Ref.
RGZ	In vitro		
	Glioblastoma U87	VEGF↓	[57]
	Lewis lung carcinoma	VEGF↓	[57]
	Pancreatic tumor AsPC-1 cells	tPA↓	[58]
	Human breast cancer cell line MDA-MB-	TIMP-1↑	[59]
	231	gelatinases↓	
	Transformed human endometrial cells	VEGF↓	[60]
	(transiently transfected Ishikawa cells)		
	Human anaplastic thyroid carcinoma cells	invasive potential↓	[61]
	MSA, IAA, ROA, K119, KOA-2		
	In vivo		
	Chick chorioallantoic membrane	Choroidal	[57]
		neovascularization↓	
	C57/BL6 xenografted with 253J B-v	Neovascularization↓	[62]
	bladder tumor cells	EC apoptosis↑	

TGZ	In vitro		
	Human non small cell lung	ELR + CXC	[63]
	cancer cells A459	chemokines↓	
	In vivo		
	C57/BL6 xenografted with 253J B-v	Neovascularization↓	[62]
	bladder tumor cells	EC apoptosis↑	

CGZ	In vitro		
	Human non-small-cell lung carcinoma	PGE_2_, COX-2↓	[64]
	A427 and A549 cell		
	Human ovarian cancer cells OVCAR-2,	VEGF, PGE_2_↓	[65]
	DISS		
	In vivo		
	BALB/c nu/nu mice xenografted with	VEGF, PGE_2_↓	[65]
	OVCAR-2 or DISS		

PGZ	In vitro		
	Renal cell carcinoma cells SMKT-R-1, -2,	VEGF, bFGF↓	[66]
	-3, -4		
	Human non small cell lung	ELR + CXC	[63]
	cancer cells A459	chemokines↓	
	Human anaplastic thyroid carcinoma cells	invasive potential↓	[61]
	MSA, IAA, ROA, K119 and KOA-2		

15d-PGJ2	In vitro		
	Renal cell carcinoma SMKT-R-1, -2, -3, -4	VEGF, bFGF↓	[66]
	Human gastric cancer	Ang-1↓	[67]
	cells MKN45		
	Human PC-3 cells	VEGF↑	[68]
	Human 5637 urinary bladder cells	VEGF↑	[68]
	Human breast MCF-7 cells	VEGF↑	[69]
	Human anaplastic thyroid carcinoma cells	invasive potential↓	[61]
	MSA, IAA, ROA, K119, KOA-2		
	Human pancreatic cancer cells BxPC-3	MMP-2, -9↓	[70]
	Transformed human endometrial cells	VEGF↓	[60]
	(transiently transfected Ishikawa cells)		
	In vivo		
	C57/BL6 xenografted with 253J B-v	Neovascularization↓	[62]
	bladder tumor cells	EC apoptosis↑	

RS5444	In vitro		
	Human anaplastic thyroid carcinoma cells	CD31↓	[71]
	DRO90-1, ARO81		
	In vivo		
	Nude mice xenografted with DRO90-1 or	CD31↓	[71]
	ARO81 tumor cells		

RS1303	In vitro		
	Human anaplastic thyroid carcinoma cells	Invasive potential↓	[61]
	MSA, IAA, ROA, K119, KOA-2		

Nimesulide	In vitro		
	Human pancreatic cancer cells BxPC-3	VEGF↑	[72]
	and MIA PaCa-2		
